# Fractured Osteochondroma: A Case Report

**DOI:** 10.7759/cureus.79478

**Published:** 2025-02-22

**Authors:** Jacob L Kraus, Akash Maheshwari, Mukul Maheshwari

**Affiliations:** 1 Radiology, Baylor College of Medicine, Houston, USA; 2 Medical School, Texas Tech University Health Sciences Center School of Medicine, Lubbock, USA

**Keywords:** fractured osteochondroma, muskuloskeletal, osteochondroma, pedunculated, pedunculated osteochondroma

## Abstract

Osteochondroma fractures, although uncommon, are a clinically significant diagnosis that should be considered in patients with acute pain about an osteochondroma following physical activity. Imaging is essential for diagnosing these fractures and guiding management. Here we present a case of a fractured pedunculated osteochondroma.

## Introduction

Osteochondromas are the most common benign bone tumors that typically arise in the lower extremities [1,2]. Osteochondromas have a male-to-female predominance of approximately two to one [1]. They can occur as focal lesions or multifocally in patients with multiple hereditary exostosis [3]. A distinguishing feature of these lesions is their cartilaginous caps which decrease in thickness after skeletal maturity is reached [1,3]. While most of these lesions are benign, there is an up to 5% risk of malignant transformation to chondrosarcoma [1]. Malignant transformation is indicated by thickening of the cartilage cap to more than 1.5 cm following skeletal maturity [4]. While benign lesions are often asymptomatic, they can lead to complications such as fractures, particularly in pedunculated lesions around the knee joint [2]. Fractures are usually triggered by physical activity and can result in pain, swelling, and reduced joint mobility [3]. Common mechanisms of fractures include direct blows to the lesions, indirect injury, or impingement from surrounding soft tissues [2,3]. Regarding treatment, observation is usually preferred, but surgical excision is considered in younger patients involved in athletic activities or those with prolonged symptoms [2]. Surgery is also preferred in cases where there is significant displacement of the fractured lesion [3]. Here we present a case of a fractured osteochondroma. 

## Case presentation

**Figure 1:**
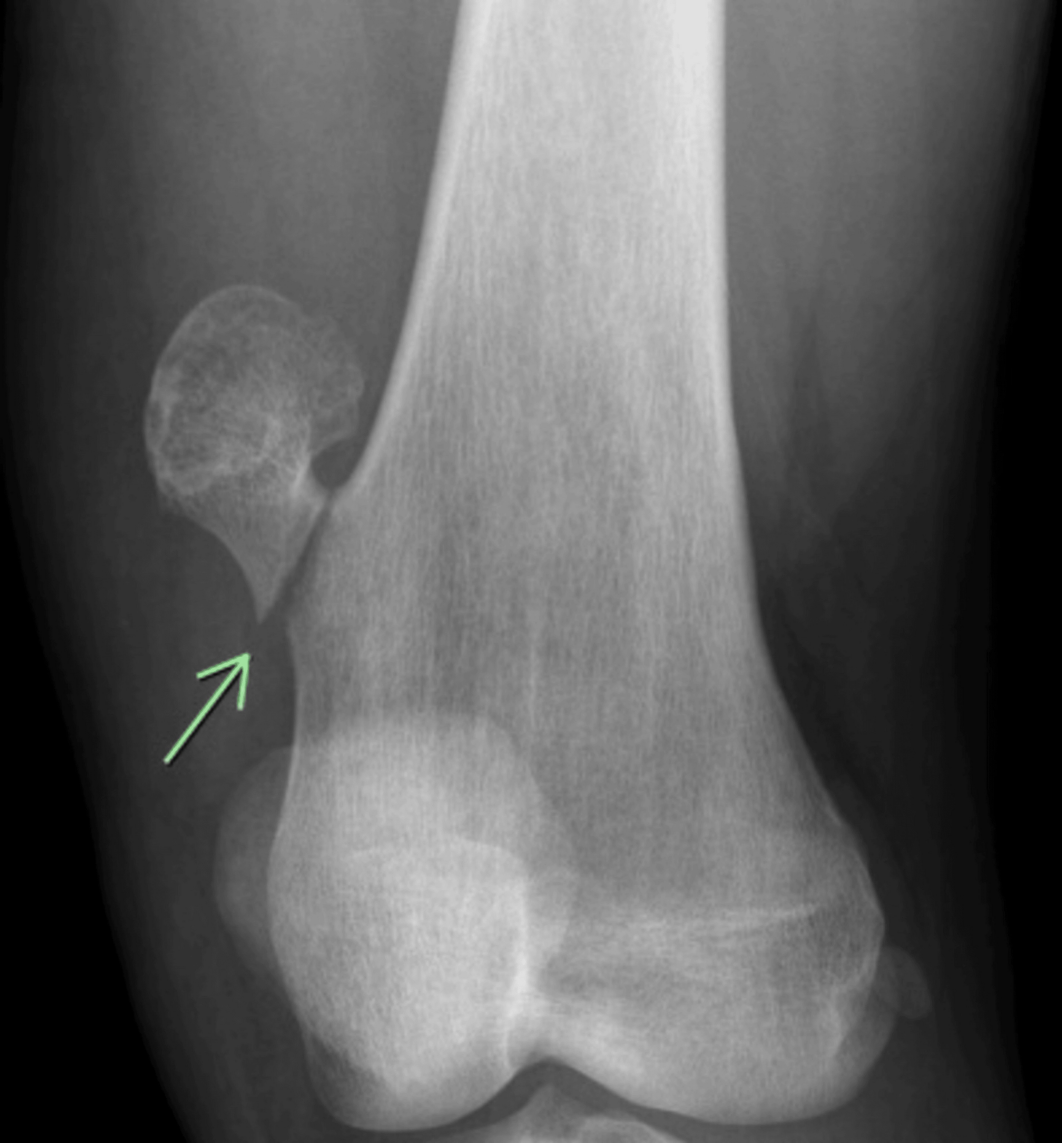
Oblique left knee radiograph showing a linear lucency through the base of a pedunculated osteochondral lesion representing a fracture.

**Figure 2:**
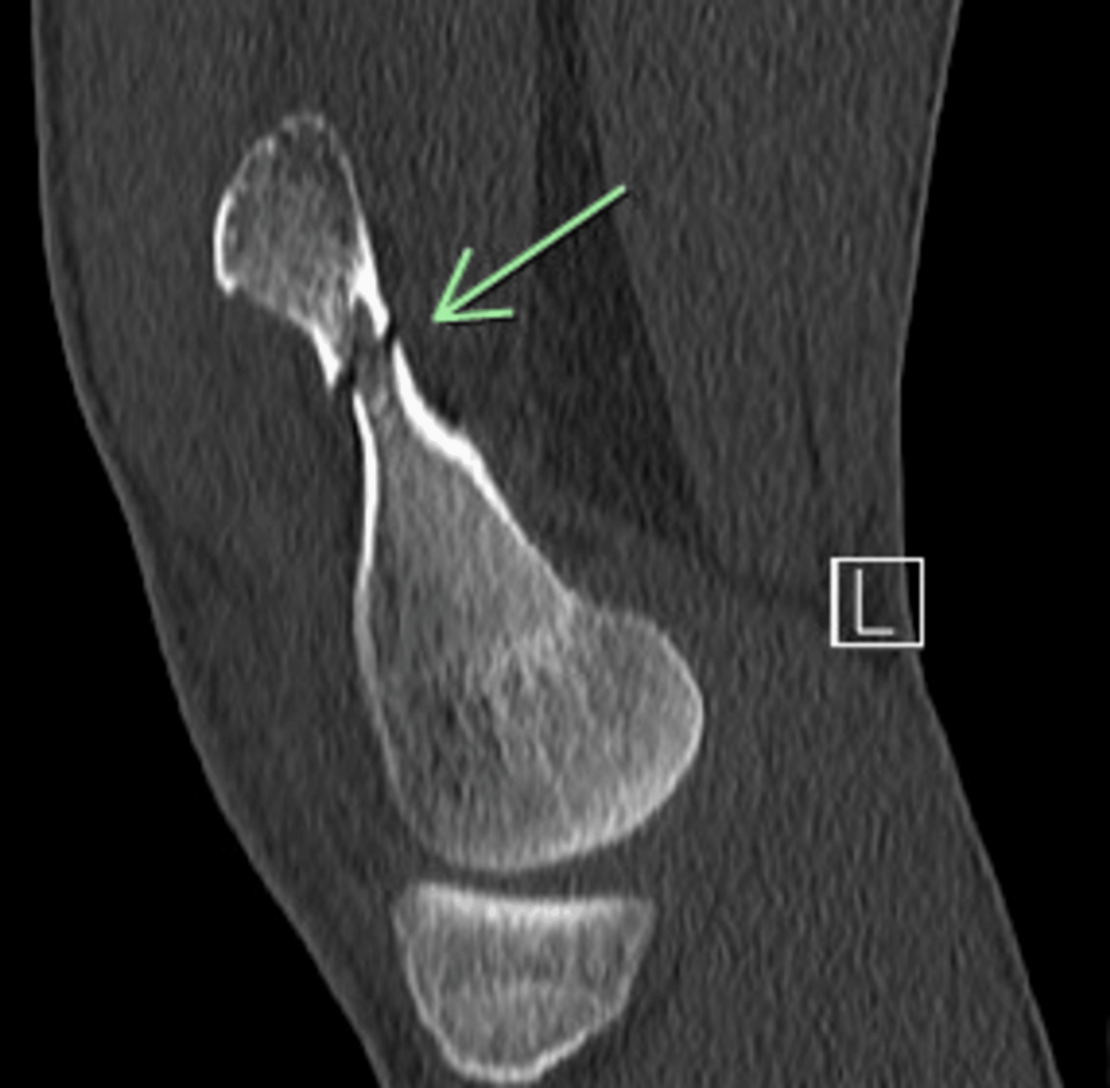
Sagittal CT showing a fracture line through the base of a pedunculated osteochondral lesion representing a fracture.

An 18-year-old male with a known left femur osteochondroma presented with sharp pain and a palpable prominence over his left inferomedial femur following an unspecified injury while playing basketball two weeks prior. The patient had full active and passive range of motion and no strength or neurologic deficits. A radiograph of the left femur was obtained for his initial workup. This showed a lucency through the base of a pedunculated osteochondral lesion which likely represented a fracture (Figure 1). A CT of the left lower extremity was then obtained which confirmed fracture of the patient’s known osteochondral lesion (Figure 2). The patient was then seen by orthopedic surgery. Given the minimal displacement of the fracture and preserved full range of motion, the patient was prescribed six weeks of left lower extremity non-weight bearing and clinical follow-up in two weeks. The patient was subsequently lost to follow-up. 

## Discussion

Osteochondromas are the most common osseous lesions, representing 20-50% of benign lesions and 10-15% of all osseous lesions as a whole [4]. Osteochondromas are cartilage-capped bony outgrowths that present as an asymptomatic cosmetic deformity or focal area of swelling [5]. They develop predominantly in young males, often before the age of 20 [6]. Lesions frequently arise from the appendicular skeleton, specifically the lower extremities [1]. Osteochondromas usually occur as solitary lesions, however 15% arise as multiple lesions secondary to hereditary multiple exostoses (HME) [7]. On imaging, osteochondromas demonstrate cortical and medullary contiguity with the associated bone of origin [8]. Osteochondromas can be described as pedunculated when arising from a thin stalk or sessile if they are broad based [6]. The cartilage cap is best identified on MRI, where it is iso to hyperintense on T2 weighted and proton density sequences [6]. Malignant transformation occurs in up to 2.5% of solitary lesions and up to 10% in patients with HME [8,9]. Features suspicious for malignant transformation include new regional pain, increased swelling, increased growth rate, and persistent growth following skeletal maturity [10]. Osteochondroma fractures, though relatively uncommon, can occur in about 5.1% of cases, particularly in pedunculated lesions around the knee joint [2]. Fractures are usually the result of physical activity. Symptoms include regional pain, edema, and reduction of neighboring joint mobility [2,3]. Treatment of osteochondroma fractures usually involves observation versus surgical excision [11]. Surgical excision may be preferred in cases of unresolved symptoms or in young athletes due to quicker return to play [3]. Rarely, untreated femoral osteochondromas can cause vascular and neurologic complications such as popliteal pseudoaneurysms, thrombosis, and nerve compression [12,13].

## Conclusions

Osteochondroma fractures, though relatively uncommon, are an important diagnosis to consider in patients with acute pain and reduced range of motion about an osteochondroma following physical activity. Imaging is essential in diagnosing fractures and guiding management, which typically involves observation but may require surgical excision in cases of persistent symptoms or in young athletes. Understanding the risk factors, imaging features, and appropriate management is essential for preventing long-term complications and ensuring optimal patient outcomes.

## References

[1] Tong K, Liu H, Wang X (2017). Osteochondroma: review of 431 patients from one medical institution in South China. J Bone Oncol.

[2] Carpintero P, León F, Zafra M, Montero M, Berral FJ (2003). Fractures of osteochondroma during physical exercise. Am J Sports Med.

[3] Futani H, Kawaguchi T, Sawai T, Tachibana T (2023). Treatment strategy of fractured osteochondroma in the young athlete’s knee. J Clin Med.

[4] Murphey MD, Choi JJ, Kransdorf MJ, Flemming DJ, Gannon FH (2000). Imaging of osteochondroma: variants and complications with radiologic-pathologic correlation. Radiographics.

[5] Motamedi K, Seeger LL (2011). Benign bone tumors. Radiol Clin North Am.

[6] Alabdullrahman LW, Mabrouk A, Byerly DW (2025). Osteochondroma. StatPearls [Internet].

[7] Bovée JV (2008). Multiple osteochondromas. Orphanet J Rare Dis.

[8] Tepelenis K, Papathanakos G, Kitsouli A (2021). Osteochondromas: an updated review of epidemiology, pathogenesis, clinical presentation, radiological features and treatment options. In Vivo.

[9] Garcia RA, Inwards CY, Unni KK (2011). Benign bone tumors--recent developments. Semin Diagn Pathol.

[10] de Souza AM, Bispo Júnior RZ (2014). Osteochondroma: ignore or investigate?. Rev Bras Ortop.

[11] Kose O, Ertas A, Celiktas M, Kisin B (2009). Fracture of an osteochondroma treated successfully with total excision: two case reports. Cases J.

[12] Göçmen S, Topuz AK, Atabey C, Şimşek H, Keklikçi K, Rodop O (2014). Peripheral nerve injuries due to osteochondromas: analysis of 20 cases and review of the literature. J Neurosurg.

[13] Miri R, Mazzaccaro D, Ziadi J (2024). Popliteal artery pseudoaneurysms in patients affected by osteochondroma. Vascular.

